# Current challenges in providing bariatric surgery in France: A nationwide study

**DOI:** 10.1097/MD.0000000000005314

**Published:** 2016-12-09

**Authors:** Sébastien Czernichow, Michel Paita, David Nocca, Simon Msika, Arnaud Basdevant, Bertrand Millat, Anne Fagot-Campagna

**Affiliations:** a Assistance-Publique Hôpitaux de Paris, Department of Nutrition, Georges-Pompidou european Hospital, Centre Spécialisé Obésité IdF Sud, Paris; b Inserm UMS 011, Population-Based Cohort Group, Villejuif; c Caisse Nationale d’Assurance Maladie des Travailleurs Salariés, Paris; d Department of Surgery, CHU Montpellier, Faculty of Medicine of Montpellier, Montpellier; e Service de Chirurgie Digestive, Centre Intégré de l’Obésité CINFO, CHU Louis Mourier (AP-HP), Colombes; f Université Paris Diderot, PRES Sorbonne Paris Cité; g Institute of Cardiometabolism and Nutrition, ICAN, Heart and Nutrition Department, Assistance-Publique Hôpitaux de Paris, Pitié-Salpêtrière Hospital; h France Sorbonne Universities, University Pierre et Marie Curie, Paris, France.

**Keywords:** bariatric surgery, obesity and healthcare expenditure

## Abstract

Bariatric surgery is a well-accepted procedure for severe and massive obesity management. We aimed to determine trends, geographical variations, and factors influencing bariatric surgery and the choice of procedure in France in a large observational study.

The Health Insurance Fund for Salaried Workers (Caisse National Assurance Maladie Travailleurs Salariés) covers about 86% of the French population. The *Système National d’Information Inter-régimes de l’Assurance Maladie* database contains individualized and anonymized patient data on all reimbursements for healthcare expenditure. All types of primary bariatric procedures (Roux-en-Y gastric bypass [RYGB] or omega loop, adjustable gastric banding [AGB], or longitudinal sleeve gastrectomy [LSG]) performed during 2011 to 2013 were systematically recorded. Surgical techniques performed by region of residence and age-range relative risks with 95% confidence intervals of undergoing LSG or RYGB versus AGB were computed.

In 2013, LSG was performed more frequently than RYGB and AGB (57% vs 31% and 13%, respectively). A total of 41,648 patients underwent a bariatric procedure; they were predominantly female (82%) with a mean (±standard deviation) age of 40 (±12) years and a body mass index ≥40 kg/m^2^ for 68% of them. A total of 114 procedures were performed in patients younger than 18 years and 2381 procedures were performed in patients aged 60 years and older. Beneficiaries of the French universal health insurance coverage for low-income patients were more likely to undergo surgery than the general population. Large nationwide variations were observed in the type choice of bariatric surgical procedures. Significant positive predictors for undergoing RYGB compared to those for undergoing AGB were as follows: referral to a center performing a large number of surgeries or to a public hospital, older age, female gender, body mass index ≥50 kg/m^2^, and treatment for obstructive sleep apnea syndrome, diabetes, or depression. Universal health insurance coverage for low-income patients was inversely correlated with the probability of RYGB.

Differences in access to surgery have been observed in terms of the patient's profile, geographical variations, and predictors of types of procedures. Several challenges must be met when organizing the medical care of this growing number of patients, when delivering surgery through qualified centers while assuring the quality of long-term follow-up for all patients.

## 1 Introduction

Bariatric surgery is now a widespread treatment option for severe and massive obesity in developed and emerging countries. The total number of procedures performed worldwide has been increasing and is now estimated to be >340,000/y, with North America and Brazil being the leading countries.^[1]^ Similar increasing trends have been observed in European countries, such as a 10-fold rise in the number of procedures since 2000 in England.^[2]^

Unsolved challenges concern the organization and funding of bariatric surgery, such as identifying the determinants pleading for 1 bariatric procedure over the other. And these determinants vary across the world and even within Europe.^[3]^ The decision process is easier in type 2 diabetic patients^[4]^ since several large randomized trials indicate some superiority for Roux-en-Y gastric bypass (RYGB) compared to longitudinal sleeve gastrectomy (LSG).

As in other countries, the current French medical guidelines require a medical management program before undergoing surgery. Eligibility is also based on body mass index (BMI) ≥40 or ≥35 kg/m^2^ with ≥1 comorbidity. Surgery before 18 years and after 60 years should be considered only on a case-by-case basis. Few nationwide studies allowing a better understanding of provided bariatric surgery have been conducted, and France provides a specific framework in which the national healthcare system covers all patients with no limitation to the supply of surgery when guidelines are respected.^[5,6]^

In the present study, we describe the trends, geographical variations in France, and factors such as differences between public and private sectors, patient characteristics, regional prevalence of obesity, comorbidities or social factors influencing bariatric surgery, and the choice in the type of procedure. We used the “National Health Insurance database” (*Système National d’Information Inter-régimes de l’Assurance Maladie* [SNIIRAM]) that collects all nationwide reimbursements for inpatient and outpatient healthcare expenditure.

## 2 Methods

### 2.1 Study design and participants

French National Health Insurance covers the entire population. The general scheme, the Health Insurance Fund for Salaried Workers (Caisse National Assurance Maladie Travailleurs Salariés), covers all French employees, as well as specific subgroups such as students, and accounts for about 86% of the population (about 58.2 million beneficiaries in 2010). Agricultural workers and farmers, self-employed, and 12 other specific schemes cover the remaining 14% of the population. The SNIIRAM database contains individualized and anonymized patient data on all reimbursements for healthcare expenditure including pharmaceutical products as well as outpatient medical and nursing care, prescribed or performed by French healthcare professionals. This database includes all hospitalizations recorded in the hospital discharge database (*Programme de Médicalisation des Systèmes d’Information*), all prescriptions, and healthcare services and procedures reimbursed together with their respective dates. Drugs are identified using their Anatomical Therapeutic Classification code, while medical and surgical procedures are identified using another specific classification. The SNIIRAM does not contain any medical information regarding prescriptions or clinical examinations, but includes data on the presence of chronic diseases eligible for 100% reimbursement of healthcare expenditure following approval by a national health insurance physician. Chronic diseases and hospital diagnoses are coded according to the International Classification of Diseases (ICD-10, http://www.who.int/classifications/icd/en). Analysis of the SNIIRAM, including the *Programme de Médicalisation des Systèmes d’Information* database, was approved by the French data protection authority (*Commission Nationale de l’Informatique et des Libertés*).

### 2.2 Inclusion criteria

All patients undergoing primary bariatric surgery procedures—gastric bypass (RYGB or omega loop), adjustable gastric banding (AGB), and LSG—with a main diagnosis of obesity in 2011, 2012, and 2013 were included in the study. The diagnosis codes for obesity in the ICD-10 were used: E66.0—obesity due to excess calories; E66.1—drug-induced obesity; E66.2—extreme obesity with alveolar hypoventilation; E66.8—other obesity; and E66.9—obesity, unspecified. ICD-10 codes for bariatric surgery procedures were the following: HFCA001, HFCC003, HFFA011, HFFC018, HFMA010, HFMC006, HFMA009, and HFMA007. A specific code for sleeve gastrectomy (SG) was introduced in September 2011; before that, LSG was coded as vertical banded gastroplasty.

### 2.3 Exclusion criteria

Other bariatric procedures such as biliopancreatic diversion were not included in the present study since only a small number of these procedures are performed in France each year (n < 100). Two patients with missing data for age were excluded. A digestive cancer was diagnosed in 75 patients, an endoscopic intragastric balloon was used in 456 patients, and 696 patients had missing data for obesity codes; all of them were excluded from the analyses.

### 2.4 Covariates

BMI was available during the hospital stay and is classified in the SNIIRAM database according to the following categories: 30 to 39, 40 to 49, and ≥50 kg/m^2^. The universal health insurance coverage for low-income patients (“*C*ouverture Médicale Universelle”) provides 100% reimbursement of health expenditures, and the data were recorded for all patients. The presence of coronary heart disease or heart failure was defined on the basis of ICD-10 codes for a chronic disease eligible for 100% reimbursement of healthcare expenditure during 2012 to 2013, or ICD-10 codes of all diagnoses coded during hospitalizations in 2009 to 2013. The presence of obstructive sleep apnea syndrome (OSAS) was determined by reimbursement for continuous positive airway pressure during the previous 12 months or hospitalization with a diagnosis of OSAS. Treatments for diabetes, asthma, chronic obstructive pulmonary disease, and depression were defined by the presence of 3 drugs’ reimbursements during the previous year.

### 2.5 Statistical analyses

Baseline characteristics were described according to gender, age, and type of surgery (AGB, sleeve, or bypass). Age- and sex-standardized rates according to the structure of the whole French population for the same year of surgery were computed in each region of France. Data are presented according to the regional number of procedures and the relative rate of a given procedure compared to the other 2 procedures. Prevalence was calculated without confidence intervals, as our estimates were based on all individuals undergoing bariatric surgery in France and not on a sample of the population.

Stepwise descending linear regressions were computed to identify predictive factors for gastric bypass compared to AGB (reference group, *P* < 0.20 for variable selection). Similar analyses were performed for SG compared to AGB. Multivariate relative risks (RRs) with 95% confidence intervals are presented. *P* values <0.05 were considered to be significant in multivariate analyses. All analyses were performed with SAS software (Cary, NJ).

## 3 Results

### 3.1 Patient characteristics and time trends

A total of 41,648 patients underwent a bariatric procedure in 2013, including 114 patients younger than 18 years (Table 1). Overall, 82.3% were females, with a mean (±standard deviation) age of 40 (±12) years and a BMI ≥40 kg/m^2^ for 68% of the population. The percentage of patients with universal health insurance coverage for low-income people undergoing bariatric surgery was higher than that of the general population after adjustment for age and gender (10.3% vs 7.4%), especially among females and teenagers. OSAS was diagnosed or treated in 29% of the population. Antihypertensive, lipid-lowering, and antidiabetic drugs were used by 26%, 12%, and 11% of patients, respectively.

**Table 1:**
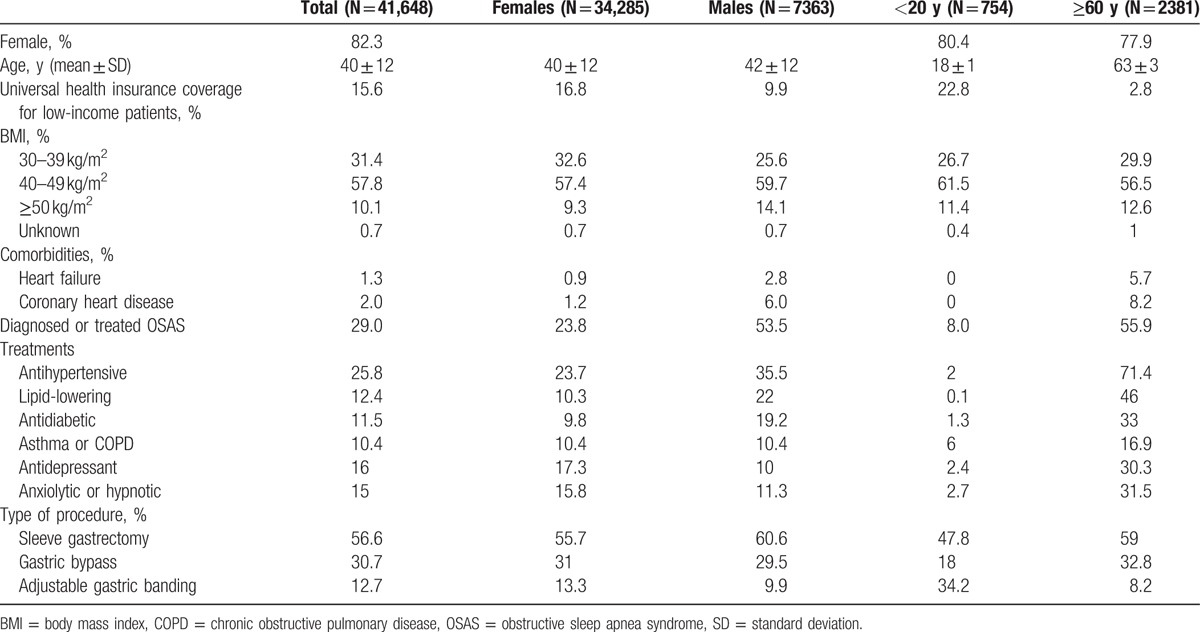
Characteristics of patients who underwent bariatric surgery in France in 2013, by age and gender.

Patients with AGB, compared to those undergoing other procedures, had a less severe medical profile with a relatively higher proportion of BMI between 30 and 39 kg/m^2^ and fewer treatments for comorbidities (Table 2). The percentage of patients with universal health insurance coverage was higher among patients treated by AGB (20%), compared to those treated by another procedure (13% and 16% for gastric bypass and SG, respectively).

**Table 2:**
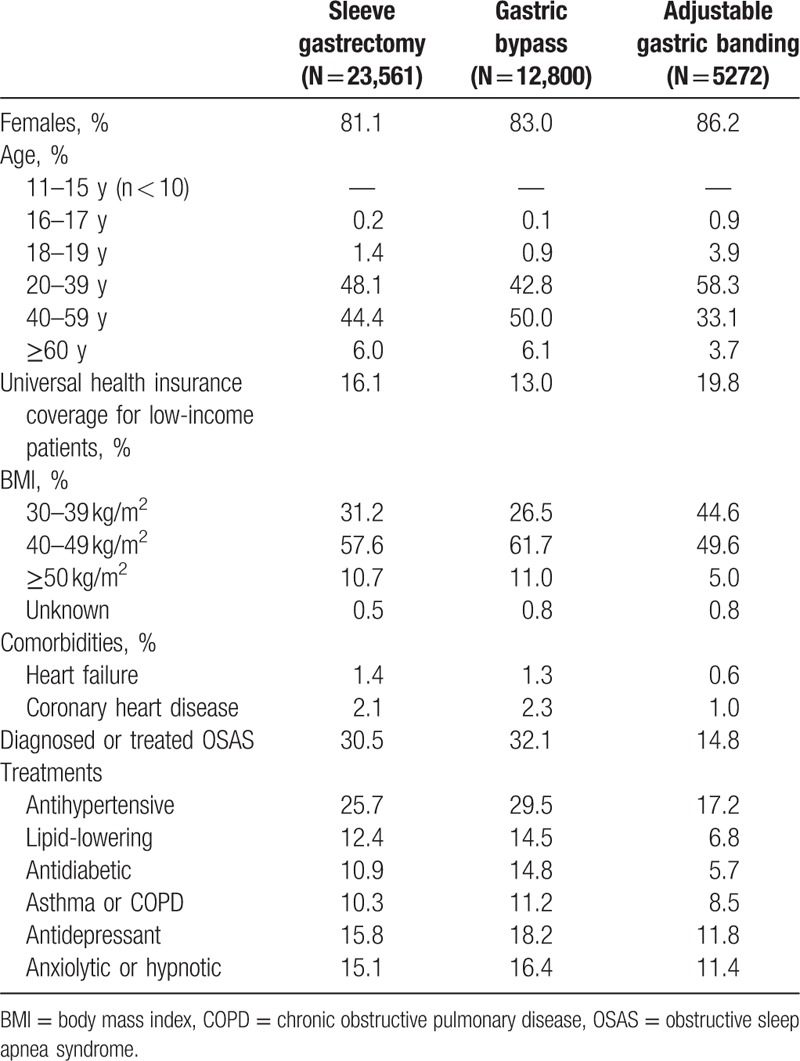
Patient characteristics by type of bariatric procedure in France in 2013.

**Figure 1:**
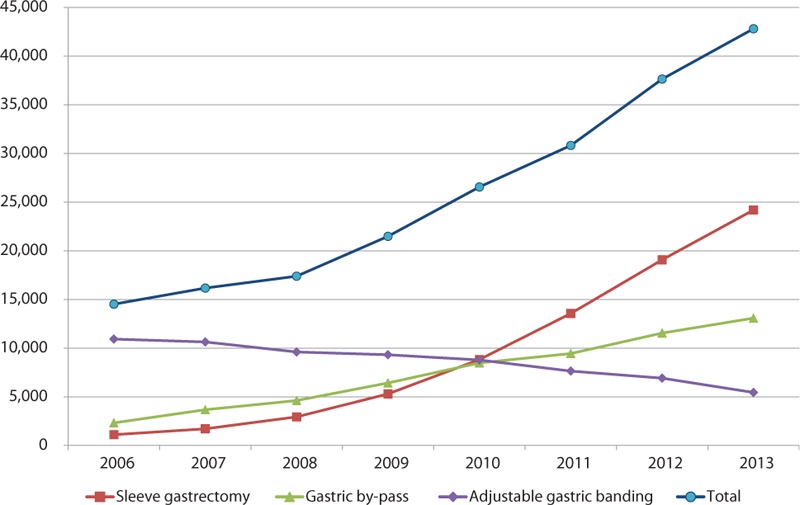
Trends in bariatric surgery in France from 2006 to 2013.

Since 2011, SG has become the most common bariatric surgery technique performed in France and AGB has become the least commonly used technique, in both males and females, and also in patients 60 years or older (Table 1; Fig. 1). SG (48%) and AGB (29%) were the most prevalent procedures in teenagers in 2013.

### 3.2 Regional variations in access to bariatric surgery

**Figure 2:**
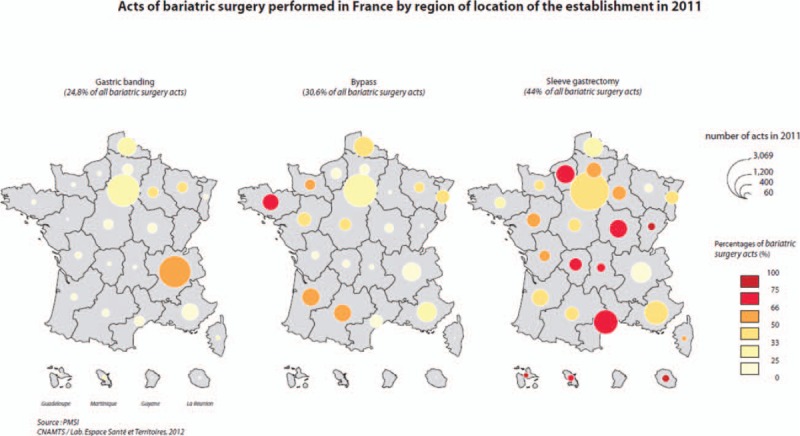
Geographical variations in access to bariatric surgery according to the 3 main procedures performed in France (adjustable gastric banding, gastric bypass, and sleeve gastrectomy). Sizes of the circles denote the number of procedures. Color codes indicate the relative proportion of each procedure compared to the other 2 procedures in the corresponding region.

Regional differences in bariatric surgery use have been observed in France (Fig. 2). More than 460 public or private hospitals perform bariatric surgery, but 175 of these centers perform <30 procedures/y (data not presented). In Fig. 2, the circle size represents the number of each procedure performed in each region, ranging from <60 to >3069/y. For example, a large number of procedures are performed in the Parisian region due to the large number of inhabitants and greater accessibility to surgery compared to less populated regions. On each map, the color of the circle reflects the relative proportion of the procedure compared to the other 2 procedures. For regions such as Paris, with a large number of procedures, the intermediate color for each procedure indicates that access to the various techniques is situated within the mean overall national range. The large red circles displaying certain techniques in regions other than Paris, such as south of France for SG, indicate that this type of bariatric surgery is more frequently performed on patients referred to bariatric surgery centers in this region compared to France as a whole. Another example of regional differences is the large number and marked preference for AGB observed in Eastern France.

### 3.3 Predictive factors influencing bariatric surgery

Multivariate linear regressions were used to identify predictive factors influencing the choice of a gastric bypass or SG procedure versus AGB (Table 3). Compared to AGB, significant positive predictors for gastric bypass or SG were as follows: referral to a center performing a large number of surgeries or to a public hospital, BMI ≥50 kg/m^2^, and treatment for OSAS. It also increases with age. The presence of universal health insurance coverage for low-income patients was inversely predictive of these types of surgery performance. Treatment for diabetes or depression and female gender were predictive of RYGB surgery only, compared to AGB.

**Table 3:**
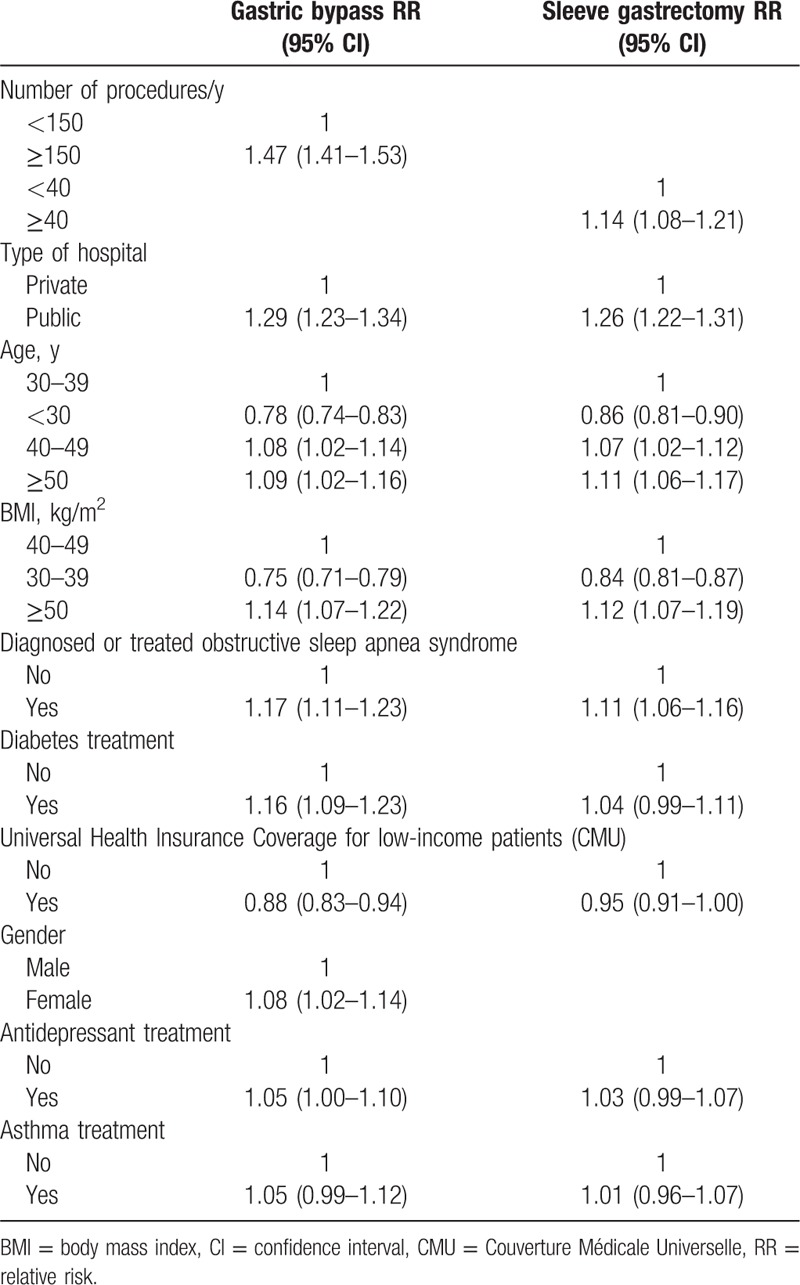
Multivariate-adjusted relative risks and 95% confidence intervals for the probability of gastric bypass or sleeve gastrectomy versus adjustable gastric banding in France at baseline in 2011 (N = 41,648).

## 4 Discussion

The present study shows that the number of bariatric procedures is steadily increasing each year in France. LSG has become the most commonly performed procedure since 2011 in both adults and teenagers, followed by RYGB in adults and AGB in younger individuals. Major regional variations in access to surgery and in the choice of procedures were observed and predictive factors for choosing RYGB or SG compared to AGB were identified, which were related to patient as well as hospital characteristics.

These changing trends in bariatric surgery are consistent with the changes observed worldwide such as in England,^[2]^ the USA and Canada,^[7,8]^ or the Asia-Pacific regions.^[9]^ Data from Europe indicate that France is now the country with the highest bariatric surgery rate alongside with Belgium and Sweden.^[3]^ This could be explained by a favorable policy context and unlimited access to bariatric surgery in France, despite the relatively modest prevalence rates of 3.1% and 1.2% for grade II and III obesity, respectively, compared to other European countries. A current specificity of France compared to other European countries is also the current and growing choice of LSG rather than other procedures. However, variations in surgery rates as well as in the choice of the procedure are also observed within France between different regions. Our data indicate that the number of bariatric procedures is not related to the prevalence of obesity in certain regions.^[10]^ For example, the prevalence of obesity in the Parisian region is only 18.5% according to the Obepi study, while the bariatric surgery rate is 8 per 10,000 inhabitants. In South-Eastern France, the prevalence of obesity is much lower, estimated at 4.3%, while the bariatric surgery rate is as high as the one observed in the Parisian region. Another example of regional differences is the large number and marked preference for AGB observed in Eastern France, which cannot be explained by a specific local health profile of the population and where the prevalence of obesity is 9.8%. Predictive factors for undergoing one type of surgery rather than another one are partially linked to patient characteristics, but also depend on the type of hospital and region. These regional differences may be attenuated over time with growing evidence in favor of a particular technique.

In other countries, such as England, a preoperative weight loss program is compulsory prior to referral to bariatric surgery. Patients who fail to comply with this mandatory preoperative program will have no access to bariatric procedure. A 2014 review from the English Health and Social Care Information Centre reported a recent decrease in the number of bariatric surgical procedures as a result of diverting patients to this preoperative program (http://www.hscic.gov.uk/catalogue/PUB13648). However, after a short period of turndown, the number of procedures could be expected to increase. Nevertheless, the long-term benefits of this mandatory program compared to other systems need to be evaluated.

Patients who undergo bariatric surgery are likely to have high universal health insurance coverage for low-income patients (15.6% and up to 23% of teenagers). Universal health insurance coverage also appears to increase the likelihood of undergoing AGB versus RYGB as well as LSG (Table 3). It has been clearly documented that obesity is more prevalent in low-income groups, but the problem of socioeconomic disparities influencing access to certain types of bariatric surgery must be addressed. A number of factors may be involved, such as the patient's preference for a less invasive procedure, the local center's experience, or even the surgeon's training. A national cross-sectional survey in 13,742 English adults indicated that patients eligible for bariatric surgery were more likely to be women, with no formal educational qualifications and retired.^[11]^ These patient characteristics in England differ slightly from those observed in the present study in France, as they were older.

Access to bariatric surgery in France is almost unlimited under the condition that bariatric surgery national guidelines are followed. One of the pitfalls of this policy is that a large number of private or public hospitals perform only a small number of procedures each year (<30) and may have limited resources for long-term nutritional follow-up.

The number of bariatric procedures is also expected to increase as an increasing number of patients will seek a second or even a third procedure after weight regain or in the context of medical or surgical complications. Having in view the large number of bariatric procedures performed each year over the past 15 years, national and regional organization of bariatric surgery now constitutes a major issue. Indeed, it needs to plan in terms of access to expert care in each region,^[12]^ individual choice of the most appropriate procedure, supply adapted to the epidemiological context, and health system funding.^[3]^ It should also specifically structure care for teenagers or elderly patients, as well as in case of reoperation.

Our study also illustrates the alarming number of procedures performed in teenagers. Since very few robust long-term data and almost no randomized evidence are available in the literature for this age group, there is an urgent need to set up national registries and long-term follow-up for this specific population. This finding also supports the urgent need for better organization of the medical management of patients of all ages. In 2012, the French Ministry of Health issued a list of 37 reference centers for the medical and surgical management of obesity. The designation of reference centers will probably reorient the demand for bariatric surgery toward high-volume centers.

The strength of the present study is that it includes all patients who underwent a bariatric procedure in France except for a few patients who did not meet the inclusion criteria. This large sample was collected across regions, allowing a detailed assessment of geographical variations of bariatric surgery. However, the present study presents a number of limitations. First, as the present study was based on a large administrative dataset, some data may be invalid or missing. However, several previous publications have demonstrated the value of using the French National Health Insurance Information System for epidemiological research in different contexts.^[13,14]^ Second, some administrative data are not sufficiently precise. For example, BMI is recorded according to 3 predefined categories: 30 to 39, 40 to 49, and ≥50 kg/m^2^, which does not allow specific analysis of patients with a BMI greater than or less than 35 kg/m^2^. Another limitation is that the present study did not assess the long-term outcomes of the various bariatric procedures and the consequences of choosing 1 technique rather than another. Lastly, our database is unable to distinguish between RYGB and omega loop gastric bypass due to the lack of a specific code for this procedure in the database.

The growth of bariatric surgery as a major treatment option for severe obesity in many countries raises a number of challenges. At first, the large and increasing number of procedures constitutes a challenge for the health system to provide the recommended postoperative follow-up. While >450,000 patients will have undergone bariatric surgery in France in 2017, organization of medical care of this growing number of patients, including well-thought access and choices of procedure, but also good quality long-term follow-up for all, constitutes a major challenge. Second, the relevance of the decision-making procedure needs to be reviewed. For instance, France has the highest bariatric surgery rate in Europe, while the prevalence of obesity is lower than that observed in the United Kingdom, Germany, Spain, or Italy.^[15]^ These discordant findings could be partially explained by the quality of financial healthcare coverage in France and no funding barriers for hospitals to develop bariatric surgery, or could reflect inadequate availability of bariatric surgery or health policies directed toward prevention and health promotion in some countries. No clear correlation has been demonstrated between bariatric surgery practices and obesity prevalence. Indeed, our data show that undergoing bariatric surgery and the choice of the procedure depend more on nonmedical factors than on patient characteristics from 1 region to another. For example, the RR of choosing RYGB or SG versus AGB is related to being referred to a public hospital. Furthermore, being covered by the universal health insurance for low-income patients will inversely influence the RR of having these 2 procedures performed, compared to AGB. Third, an alert should be raised concerning performance of bariatric surgery to teenagers and patients 60 years or older, as bariatric surgery rates are increasing in these age groups despite the limited long-term prospective data in these extreme age groups. This supports the need for revision of clinical guidelines and for national monitoring of this surgical practice.
