# Current challenges in providing bariatric surgery in France: A nationwide study: Erratum

**DOI:** 10.1097/MD.0000000000006510

**Published:** 2017-03-24

In the article “Current challenges in providing bariatric surgery in France: A nationwide study”,^[1]^ which appeared in Volume 95, Issue 49 of *Medicine*, one of Dr. Czernichow's affiliations was omitted. Dr. Czernichow is also affiliated with the University Paris Descartes, Paris, France.
